# 486. Phenotypic Clusters of Clinical Presentation and Severity Among Children with Multisystem Inflammatory Syndrome — United States, February 2020–October 2022

**DOI:** 10.1093/ofid/ofad500.556

**Published:** 2023-11-27

**Authors:** Kevin Ma, Anna R Yousaf, Katherine N Lindsey, Michael Melgar, Allison D Miller, Ami B Shah, Michael J Wu, Angela P Campbell, Laura D Zambrano

**Affiliations:** CDC, Atlanta, Georgia; Centers for Disease Control and Prevention, Atlanta, GA; Centers for Disease Control and Prevention, Atlanta, GA; Centers for Disease Control and Prevention, Atlanta, GA; Centers for Disease Control and Prevention, Atlanta, GA; General Dynamics Information Technology, Atlanta, Georgia; Centers for Disease Control and Prevention, Atlanta, GA; Centers for Disease Control and Prevention, Atlanta, GA; Centers for Disease Control and Prevention, Atlanta, GA

## Abstract

**Background:**

Multisystem inflammatory syndrome in children (MIS-C) is an uncommon but severe hyperinflammatory syndrome occurring weeks after SARS-CoV-2 infection. Presentation can vary and overlap with other conditions, including acute COVID-19 and Kawasaki disease. Identifying clusters of MIS-C phenotypes informs efforts to reduce misclassification, characterize pathogenesis, and tailor treatments. Here we describe clusters of presentation using the largest collection of U.S. MIS-C cases to date.

**Methods:**

We analyzed 9131 MIS-C cases reported to CDC from 55 U.S. health departments; onset dates ranged from February 2020–October 2022. Thirty-one dichotomous clinical variables were selected for clustering after excluding ones with high (≥ 15%) missingness or rare (≤ 10%) or high (≥ 90%) prevalence. We excluded 595 cases with ≥ 5 missing variables and conducted multiple imputation on the remaining 8536 cases. Latent class analysis (LCA) was run using the *poLCA* R package; the number of clusters was selected based on decreasing information criteria and automated variable selection.

**Results:**

LCA identified five clusters, two of which were characterized by distinct organ system involvement: a respiratory symptom-driven cluster primarily affecting older children (N = 483 [5.7% of cases]; median age 14.4 years), and a mucocutaneous symptom-driven cluster primarily affecting young children (N = 1956 [22.9%]; median age 5.8 years) (Fig. 1). The remaining three clusters delineated a spectrum of clinical severity, with clinically mild and moderate clusters with infrequent shock (N = 2566 [30.0%] and N = 1650 [19.3%], respectively), and a severe cluster (N = 1881 [22.0%]) with shock and ICU admission for nearly all cases (Fig. 2). The proportion of cases belonging to this clinically severe cluster decreased over time as proportions of the mild/moderate clusters increased (Fig. 3). The case fatality ratio was highest in the respiratory (5.0%) and clinically severe clusters (1.6%) compared with other MIS-C cases (0.2%; P < 0.001).

**Figure 1.**
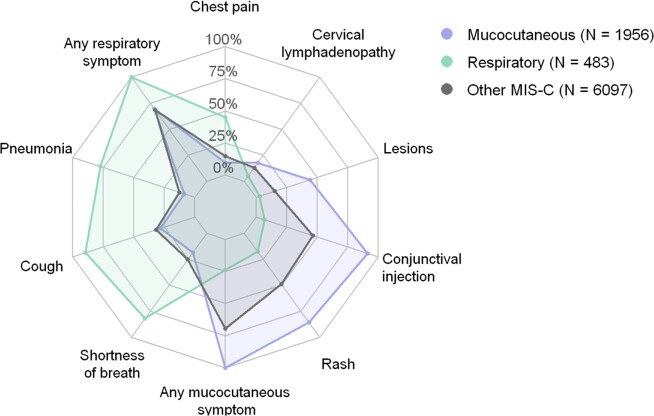
Prevalence of respiratory and mucocutaneous/dermatologic symptoms for the respiratory and mucocutaneous clusters compared to all other MIS-C cases.

**Figure 2.**
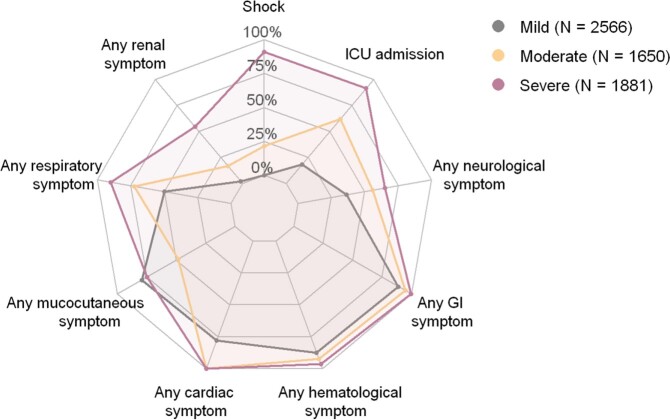
Prevalence of organ system involvement, shock, and ICU admission for the three severity-defined clusters.

**Figure 3.**
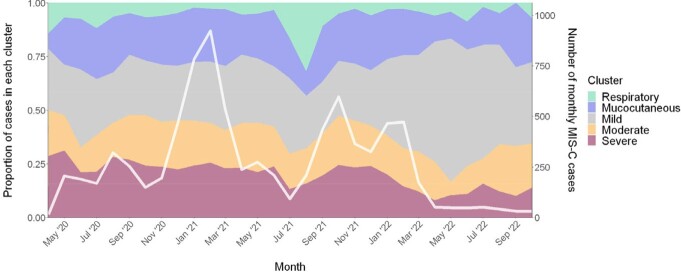
Proportions of MIS-C cases by LCA-inferred cluster and month (primary y-axis); number of monthly MIS-C cases (secondary y-axis).

**Conclusion:**

MIS-C cases reported to CDC national surveillance clustered into five groups with distinct symptoms and severity. Use of MIS-C phenotypic clusters in future studies may inform treatment decisions and assist with prognosis.

**Disclosures:**

**All Authors**: No reported disclosures

